# Efficacy of Nerve Root Block for the Treatment of Lumbar Spinal Canal Stenosis in Adults Older Than 80 Years of Age

**DOI:** 10.7759/cureus.24863

**Published:** 2022-05-09

**Authors:** Mamiko Sakai, Akihiko Inokuchi, Ryuta Imamura, Teiyu Izumi, Masatoshi Yamamoto, Masakazu Yoshimoto, Yu Soejima, Kimitaka Nakamura, Takahiro Hamada, Takeshi Arizono

**Affiliations:** 1 Department of Orthopaedic Surgery, Kyushu Central Hospital of the Mutual Aid Association of Public School Teachers, Fukuoka, JPN

**Keywords:** radiculopathy, older adults, conservative treatment, lumbar spinal canal stenosis, selective nerve root block

## Abstract

Background: Patients with advanced lumbar spinal canal stenosis (LCS) often prefer non-operative treatment owing to decreased physiological function and comorbidities. Although the therapeutic value of selective nerve root block (SNRB) for LCS is confirmed, there are few reports of its effectiveness in the elderly. We investigated the efficacy of SNRB for LCS in patients over 80 years of age.

Methods: The subjects were 112 patients aged over 80 years (mean age: 84 years; 45 men and 67 women ) with medication-resistant LCS without cauda equina syndrome who underwent SNRB. Cases with acute-onset lumbar disc herniation were excluded. We retrospectively investigated and compared the presence or absence of surgery, effect of SNRB, number of procedures, duration of disease, and magnetic resonance imaging findings. Patients who could avoid the surgery by SNRB were defined as the effective group. Patients whose symptoms were not relieved by SNRB and who underwent surgery and those whose symptoms were not relieved but who continued conservative treatment were defined as the ineffective group. A total of one to seven SNRBs were performed in both groups, and the same spine surgeon performed the entire procedure from SNRB to surgery.

Results: There were 86 nonoperative patients (69 effective cases) and 26 operative patients; the overall rate of effectiveness was 61% (69/112 patients). The area of the spinal canal at the responsible level was 108.63 mm^2^ in the effective group compared with 77.06 mm^2^ in the ineffective group. This was significantly narrower in the ineffective group (p=0.0094). There was no significant difference in the duration of illness, number of blocks, or hernia complication rate between the groups. No patient experienced severe neuralgia that may have been caused by neuropathy during SNRB.

Discussion: Our outcome showed that more than 60% of older patients with LCS showed symptomatic improvement with SNRB. SNRB can be performed relatively safely in the elderly and appears to be a favorable treatment option for older patients with various risks, such as poor general condition.

Conclusions: Multiple sessions of SNRB may provide older patients with symptomatic improvement and may be an option for treatment.

## Introduction

Lumbar spinal canal stenosis (LCS), which is caused by degenerative or age-related changes such as yellow ligament thickening and intervertebral foraminal stenosis, is reported to be more common in the elderly, with a particularly high incidence in people older than 80. Patients with LCS of the radiculopathy and mixed types often also have symptoms such as radicular pain, numbness, and intermittent claudication, which can affect patients’ quality of life and limit their activities [1]. Recently, the number of spinal surgeries has been increasing, and especially for older patients over 80 years of age the most common surgical condition is LCS. Although immediate surgical intervention is necessary when accompanied by acute lower limb paralysis or bladder or rectal disorders, in the absence of such "red flags", the effectiveness of surgical treatment is controversial [2]. In addition, physiological function declines with age, and the incidence of comorbidities, such as cardiovascular disease and renal dysfunction, increases. The risk of perioperative complications in patients with comorbidities is high, and the risk increases with age. Therefore, even patients with advanced LCS often request or are required to choose conservative therapy.

Non-operative treatment comprises rest, muscle relaxants, non-steroidal anti-inflammatory drugs, and physical therapy. Selective nerve root block (SNRB) is an option when symptoms persist or when patients are not suitable for surgery. The therapeutic value of SNRB for lumbar spinal stenosis is accepted [1,3]. SNRB can be performed at multiple sites, including the cervical and lumbar spine, and can reduce pain in patients with severe pain. However, there are scattered reports of adverse events occurring with SNRB. There are also reports of higher complication rates in patients who have received several injections compared with those who have received only one injection [4], and to the best of our knowledge, there have been no reports of the efficacy of repeated SNRBs. In addition, there are few reports of avoidance of surgery by SNRB in the elderly. In this study, we investigated the efficacy of SNRB in older patients (> 80 years of age) with LCS.

## Materials and methods

**Figure 1:**
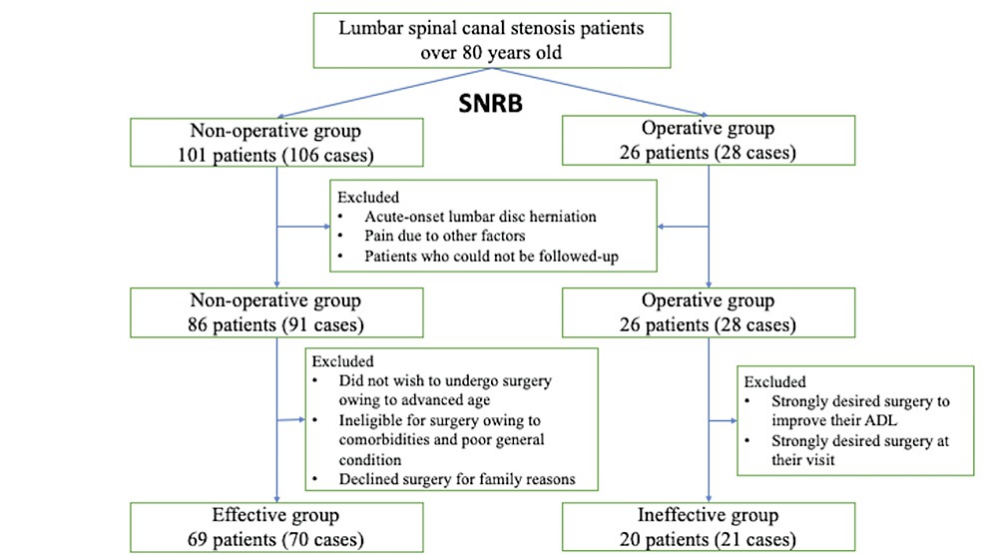
Patient flowchart SNRB: selective nerve root block, ADL: activities of daily living

A total of 127 patients who visited our hospital between 2010 and 2020 with LCS and who underwent SNRB were included in this study. LCS was diagnosed on the basis of clinical symptoms and imaging findings. Patients with acute-onset lumbar disc herniation, trauma, cauda equina syndrome, pain due to other factors, such as polymyalgia rheumatica, peripheral vascular circulatory disorders, and psychological factors, and patients who could not be followed up owing to non-attendance or transfer of doctor after SNRB were excluded. After excluding these patients, 112 patients were included in the study (Figure 1). The mean age of the patients was 84 years (range: 80-92 years); 45 were men and 67 were women.

We clarified the presence or absence of surgery, number of blocks performed, block effect, magnetic resonance images, presence or absence of complications, and disease duration by clinical records retrospectively. The magnetic resonance images were examined for the area of the spinal canal at the responsible level and for the presence or absence of herniation. The area of the spinal canal was calculated as the average of three measurements at the same level. For patients with visual analog scale (VAS) records, those whose scores decreased over time were defined as those with pain reduction; for those without VAS records, pain reduction was evaluated based on patient statements and their activities of daily living (ADL) improvements noted in the patient's medical record at the next visit. Of the nonoperative patients, those who achieved pain relief with SNRB were defined as the effective group, excluding those who did not wish to undergo surgery owing to advanced age, those who were ineligible for surgery owing to comorbidities and poor general condition, and those who declined surgery for family reasons. Of the patients who underwent surgery, the ineffective group comprised those who underwent surgery owing to inadequate efficacy of SNRB, excluding those who strongly desired surgery to improve their ADL even though SNRB had some efficacy, and those who strongly desired surgery at their visit. We compared the results of each investigated item between the effective group and the ineffective group. This study was approved by Kyushu Central Hospital Review Board on Clinical Research Plans (approval number 287) and conducted at Kyushu Central Hospital, Fukuoka, Japan.

Technique

**Figure 2:**
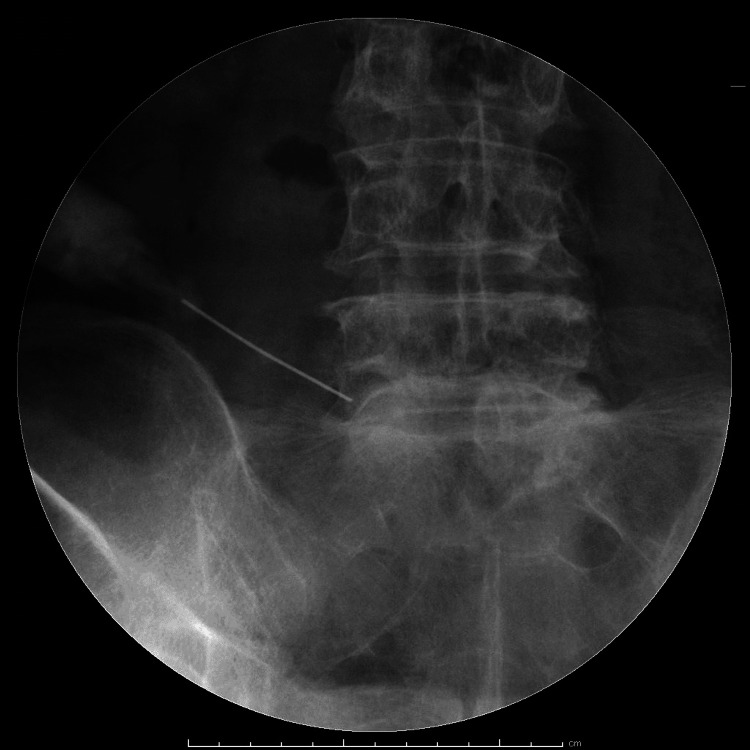
Fluoroscopic image at selective nerve root block (SNRB)

SNRB was performed by the same spine surgeon. The patient was placed in the prone position under fluoroscopy and received an injection of 1% mepivacaine. Although pain reproduction was not always confirmed, the drug was injected after confirming that the needle tip was in the optimal position (Figure 2). The procedure was performed one to seven times in all patients. We performed SNRB at one- to four-week intervals, depending on the patient's symptoms.

Statistical analysis

Data for both groups were confirmed to be normally distributed, and results were compared using t-tests for each category. Analyses were performed using Excel (Microsoft, Redmond, WA, USA). P<0.05 was considered statistically significant.

## Results

Of the 127 patients who underwent SNRB, 112 met the inclusion criteria; 86 patients (91 cases) were nonoperative, and 26 patients (28 cases) were operative. Of the non-operative cases, 69 patients (70 cases) comprised the effective group, and of the surgically treated cases, 20 patients (21 cases) comprised the ineffective group (Figure 1). Of the 112 evaluable patients, 69 patients comprised the effective group and 61% were able to avoid surgery, with SNRB. The mean age was 85.0 years in the effective group and 83.2 years in the ineffective group.

Comparing each category, the spinal canal area at the responsible level was 108.63 mm^2^ in the effective group and 77.06 mm^2^ in the ineffective group, and the ineffective group had a significantly narrower spinal canal area than the effective group (p=0.0094) (Table 1). The duration of disease, number of blocks, and lumbar disc herniation rate were 8.43 months/18.95 months (p=0.112), 3.03 times/3.19 times (p=0.697), and 38.2%/47.6% (p=0.449), in the effective/ineffective groups, respectively, with no significant difference.

**Table 1:** Results of each category between the effective and ineffective groups

	Effective group (n=69)	ineffective group (n=20)	p-value
Spinal canal area at the responsible height (mm^2^)	108.63	77.06	0.0094
Duration of disease (months)	8.43	18.95	0.112
Number of blocks (times)	3.03	3.19	0.697
Lumbar disc herniation rate (%)	38.2	47.6	0.449

Although one of the patients in the effective group had a symptom relapse six months after the initial symptom relief, the SNRB again relieved the symptoms. Thereafter, no symptom recurrence was observed. Of the ineffective group, three patients relapsed two to six months after symptom relief with SNRB and required surgical treatment. Since such patients were included in the ineffective group, the effective group in this study did not include those who required surgical treatment owing to relapse of symptoms after SNRB. There were no complications, such as residual neuralgia, hematoma, or infection, after SNRB in all patients.

## Discussion

In this study, we investigated the efficacy of SNRB in the treatment of LCS in patients older than 80 years of age. As one of the options for conservative therapy, the efficacy of SNRB has been reported often [5]. Kannan et al. investigated the efficacy of SNRB in patients with radiculopathy who continued to have a VAS score even after medication [1]. Seventy-six patients underwent SNRB and 35 patients subsequently required surgery; 54% of the patients were able to avoid surgery, with SNRB. In this study, 61% of the patients were able to avoid surgery, which provides effects similar to those of SNRB.

Regarding the prognostic factors for conservative treatment of LCS, lumbar kyphosis, range of motion, spinal canal area, and severe intermittent claudication have been reported previously [6-8]. In the present study, we found that the spinal canal area at the responsible level was significantly narrower in the ineffective group. Tadokoro et al. investigated the outcome of older patients undergoing conservative treatment and the factors affecting conservative treatment [9]. The authors reported that the rate of improvement in ADL with conservative treatment was lower in patients with complete contrast blockage at the responsible level on myelography. As in the present study, the effect of conservative therapy is poor in cases with a significantly narrowed spinal canal area, and conservative therapy may have therapeutic limitations in these cases.

In the present study, the duration of disease was not significantly different between the effective and ineffective groups; however, the p-value was low, at 0.112, and the duration of disease was 8.43 months and 18.95 months, respectively. Although there was no significant difference, the difference in disease duration was large, suggesting that the longer the disease duration, the less successful the non-operative therapy tends to be. As the number of cases increases, there will likely be a significant difference in the duration of disease.

Mallinson et al. reported that 69.1% of patients experienced pain relief within seven days after SNRB [3]. In contrast, Ko et al. investigated VAS scores over time after SNRB and reported that the pain reduction rate was highest at two weeks and that the rate decreased gradually thereafter [10]. In this study, the average number of times that SNRB was performed was three, and the efficacy rate was 61%. Two weeks after a single nerve root block was performed, the pain reduction rate was reported to decrease, and it is possible that multiple SNRBs may contribute to greater symptom relief. However, there have been no reports on the effects of multiple nerve root blocks, and this study did not reveal data to support the optimal frequency.

There have been reports of adverse events after SNRB. Manchikanti et al. reported minor complications after SNRB, such as focal hemorrhage and nerve root irritation symptoms in 3162 of 15,645 patients [11]. Stalcup et al. reported minor complications in 98 of 1777 patients who underwent SNRB [4]. The symptoms were mild and comprised transient lower extremity weakness, dizziness, and persistent pain, and the incidence was higher in patients who underwent multiple nerve root blocks. Although multiple nerve root blocks may lead to adhesions around the nerve root and increase the risk of complications, no complications were observed in any patient, and all were over 80 years old. Considering our data, SNRB can be performed relatively safely, even in the elderly.

There are several limitations to this study. First, the number of cases was small. As mentioned above, increasing the number of cases may reveal a significant difference between effective and ineffective groups, and further research is needed. Second, we were unable to score pain improvement measures such as the VAS in several of our cases. The older patients were the study population in this study, and it was difficult to match the VAS with verbal pain improvement and to reproduce the assessment by the VAS. It was possible to adequately follow the transition of symptoms through the statements of patients and family members in the medical record. Finally, the follow-up rate was low. It is true that not all patients who have improved have been followed up. Some patients may not have been able to see the doctor due to comorbidity worsening, and some may have refrained from seeing the doctor over the past few years due to COVID-19. To address this issue, we could have considered performing follow-up surveys by phone.

## Conclusions

In this study, SNRB was effective in more than 60% of older patients with LCS. The therapeutic effect of SNRB may be lower in cases of advanced LCS and in those with a long disease duration. SNRB may be a relatively safe treatment option for older patients with various perioperative risks.
